# Ultrasound-Mediated Atom Transfer Radical Polymerization (ATRP)

**DOI:** 10.3390/ma12213600

**Published:** 2019-11-02

**Authors:** Izabela Zaborniak, Paweł Chmielarz

**Affiliations:** Department of Physical Chemistry, Faculty of Chemistry, Rzeszow University of Technology, Al. Powstańców Warszawy 6, 35-959 Rzeszów, Poland; i.zaborniak@stud.prz.edu.pl

**Keywords:** external stimulus, ultrasound, mechanically-induced ATRP, ultrasonication-induced ATRP

## Abstract

Ultrasonic agitation is an external stimulus, rapidly developed in recent years in the atom transfer radical polymerization (ATRP) approach. This review presents the current state-of-the-art in the application of ultrasound in ATRP, including an initially-developed, mechanically-initiated solution with the use of piezoelectric nanoparticles, that next goes to the ultrasonication-mediated method utilizing ultrasound as a factor for producing radicals through the homolytic cleavage of polymer chains, or the sonolysis of solvent or other small molecules. Future perspectives in the field of ultrasound in ATRP are presented, focusing on the preparation of more complex architectures with highly predictable molecular weights and versatile properties. The challenges also include biohybrid materials. Recent advances in the ultrasound-mediated ATRP point out this approach as an excellent tool for the synthesis of advanced materials with a wide range of potential industrial applications.

## 1. Introduction

Reversible deactivation radical polymerization (RDRP) is the rapidly developing method in preparation of functional and specialized polymeric materials. The rapid advent of the field of controlled synthesis created the opportunity for a polymer scientist to access to materials characterized by low dispersity and predominant molecular weight, and most importantly, the possibility to receive different topology and architecture—an essential aspect from the application point of view. All the features make up the excellent toolbox for the preparation of advanced materials with desired and predictable functions and properties [1,2,3,4,5]. Among all the controlled radical polymerizations, atom transfer radical polymerization (ATRP) deserves special attention. Compared to conventional free radical polymerization, the step of radical generation is reversible and occurs by a dynamic redox mechanism. In ATRP, the radicals are obtained by reaction between a dormant (macro)molecular species/alkyl halide and an activator, metal complexes in their lower oxidation state, predominately. As a result of the reaction both propagating radicals and deactivators, higher oxidation-state transition metal complexes are formed. This state is reversible due to the possibility to re-form the activator and the dormant species by reaction between the deactivator and the propagating radical. The addition of monomer(s) to the propagating radicals leads to polymer chain growth [5,6,7]. Focusing on the latest achievements in this technique, on top of the typical characteristics for controlled radical approaches, i.e., precisely defined structure proven by narrow molecular weight distribution and high conformity of theoretical and apparent molecular weights [8,9,10], and thus preserved chain-end functionality [11,12,13]—ATRP enables to receive increasingly on complex structure in a well-controlled manner. The recent advances include, e.g., ultra-high polymer structures [14,15,16], star-like [5,11,17,18,19] and hybrid materials [20,21,22,23] with a wide range of potential industrial applications. Initially developed “normal” ATRP was characterized by relatively high catalyst loading of up to 10,000 ppm [24] (molar ratio between catalyst and monomer) to overcome the increase in concentration of the deactivator due to the termination reactions, which led to a slow-down in the rate of the reaction [24]. The breakthrough in ATRP was the development of techniques with regeneration of the catalyst complex in the form of an activator, significantly reducing the catalyst concentration to low parts per million (ppm). The primary factor constituting the driving force in this ATRP approach is an additional redox cycle created by a reducing agent [6,25,26,27]. These methods are characterized by the continuous regeneration of activators (complexes in the lower oxidation state, Cu^I^) from deactivators (complexes in the higher oxidation state, Cu^II^) due to use a chemical agent [28,29,30,31,32] or external stimuli [33,34,35,36], significantly reducing the need for high catalyst concentrations. The chemical reducing agent methods include radical initiators in continuous activator regeneration (ICAR) ATRP [37], reducing agents in activators regenerated by electron transfer (ARGET) ATRP [30], and zerovalent metals in supplemental activator and reducing agent (SARA) ATRP [29]; meanwhile, the externally controlled are the electrochemically-mediated ATRP (*e*ATRP) [8,34,35], photochemically-mediated ATRP (photo-ATRP) [33,36,38,39], and the primary subject of this review—mechanically induced ATRP (mechano-ATRP) [40,41,42] and ultrasonication-mediated ATRP (sono-ATRP) [12,14,43]. The latest considerations in the range of reducing factors revolve around external stimuli as a reducing agent. This solution avoids the use of chemical reducing agents in the reaction setup, and therefore, eliminates undesired by-products. Moreover, it allows for temporal control over the polymerization processes instead of chemical reducing agents [12,17,22,39,41].

From the last three years, sonication was considered as a predominant factor in the context of external stimuli in ATRP. Considering other ATRP approaches, especially those characterized by low ppm catalyst loading, an ultrasonication-mediated technique has measurable benefits: (i) as an externally controlled method, it enables control of the polymerization rate by switching ultrasonication on/off—an undoubted advantage in the preparation of polymers with predictable molecular weights; (ii) compared to chemical reducing agents methods, the recently developed sono-ATRP completely eliminates additional chemical compounds (sono-ATRP) or reduces the chemicals, which interfere with reactants and cause the production of by-products (mechano-ATRP); (iii) from an industry point of view, it is facile to use and scale-up; (iv) additionally, the fact that the both hydrophobic (organic and miniemulsion medium) and hydrophilic (aqueous medium) monomers are able to polymerize indicates the versatility of this approach. Sonochemistry covers the application of ultrasound agitation in the form of ultrasound-induced physical and chemical factors to a chemical process [44]. It is generated from the mechanical effect of acoustic cavitation on liquid medium resulting in the growth, formation and implosive collapse of bubbles in liquids followed by the formation of extremely high temperature and pressure conditions [45]. Considering RDRP, both reversible addition-fragmentation chain-transfer polymerization (RAFT) [46,47] and ATRP [12,40,41,42,43,48,49,50,51,52,53] successfully applied ultrasonication as an external stimulus for promoting polymerization reactions. In the field of controlled polymer synthesis, the ultrasound-mediated effect can be divided for physical sonochemistry and mechanochemistry [45,54]. The first one is connected with physical effects from collapsing cavitation bubbles, leading to the efficient production of reactive radical species, which are then used in a wide range of radical-based reactions [45,54,55,56]. Meanwhile, the mechanochemistry arises from the strong shear gradients produced by bubble collapse. Created forces can cleave covalent bonds, and alternatively, produce an electric charge in response to pressure changes with the use of piezoelectric materials [45,54,57,58]. ATRP uses the both of those approaches. It is considered in two different ways: mechano-ATRP (ultrasonication as a mechanical force to induce an electric charge in response to an applied mechanical strain with the use of piezoelectric) [40,41,42,48,49,50] and sono-ATRP, where the driving force of an activator’s regeneration are hydroxyl radicals in aqueous media [12,43,51] and reactive radical species from solvent/ligand/additional reagents in organic solvent [52,53]. The range of frequencies used in ATRP covers mainly low frequencies (20–40 kHz); however, the higher frequencies (490 kHz) have been also investigated.

The main goal of the review is to present the recent advances in the use of ultrasonication in ATRP as an external stimulus, as the most rapidly developing ATRP approach in the last few years. It summarizes the present state-of-the-art, covering ultrasound as a mechanochemical and sonochemical solution, focusing on mechanistic aspects and synthetic procedures in the preparation of functional polymers. The review also introduces future perspectives of using ultrasound agitation in controlled polymerization techniques.

## 2. Mechanically Controlled ATRP

**Figure 1:**
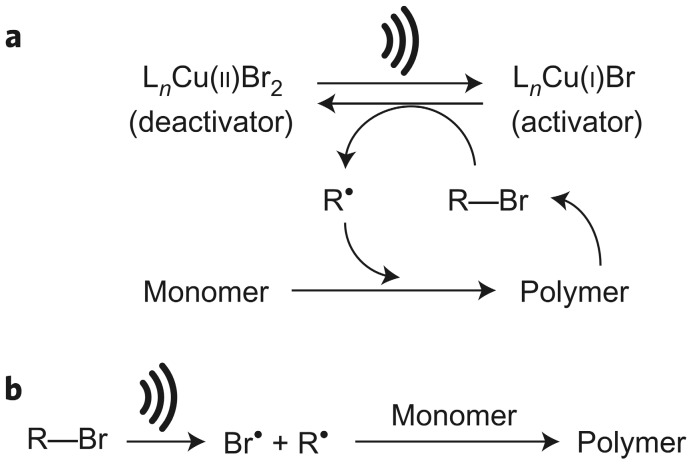
Two potential routes for the initiation of polymerization process by ultrasound agitation: (**a**) reduction of deactivator by the piezocatalytic effect resulting in the formation of an activator followed by radical generation, and thus growing of the polymer chain; (**b**) sonication-mediated generation of radicals, directly initiating a radical polymerization [48]. Reprinted with permission from Springer Nature. Copyright 2016.

The first consideration about sonication as a driving force in ATRP is focused on mechano-induced electron transfer (MET) determined by the transduction of a mechanical stimulus to an electrical signal [59,60]. In this context, piezoelectric nanoparticles (NPs) as electron transfer agents are used. This follows from the commonly known piezoelectric effect described as the induction of an electric charge in response to an applied mechanical strain; e.g., ultrasonication [44]. The recent advances and clear evolution in mechanically controlled ATRP (mechano-ATRP) approach are summarized in Table 1. The potential of this effect was for the first time used as an externally controlled ATRP approach by Mohapatra et al. in 2016 [48]. Ultrasonic agitation in connection with barium titanate nanoparticles (BaTiO_3_ NPs) was used to mechanochemically reduce a deactivator (Cu^II^) to an activator (Cu^I^) species, and thus create the possibility to initiate catalyst-mediated polymerization of *n*-butyl acrylate (BA). The choice of BaTiO_3_ was motivated by the reports proving the large electrochemical potential revealed by this transducer under the influence of ultrasound [61,62]. That points out an ultrasonic agitation as an external stimulus creating a sufficient localized potential to reduce Cu^II^ catalyst complex. Two potential mechanisms for mechanochemically-mediated, controlled radical polymerization were proposed, including (a) reduction of deactivator catalytic complex due to the BaTiO_3_-mediated piezocatalytic effect resulting in the formation of activator followed by radical generation, and thus growing of the polymer chain; (b) transfer of an electron from the BaTiO_3_ particle to another component of the sonicated polymerization mixture resulting in generation of radicals by sonication, which directly initiate a radical polymerization (Figure 1) [48].

**Figure 2:**
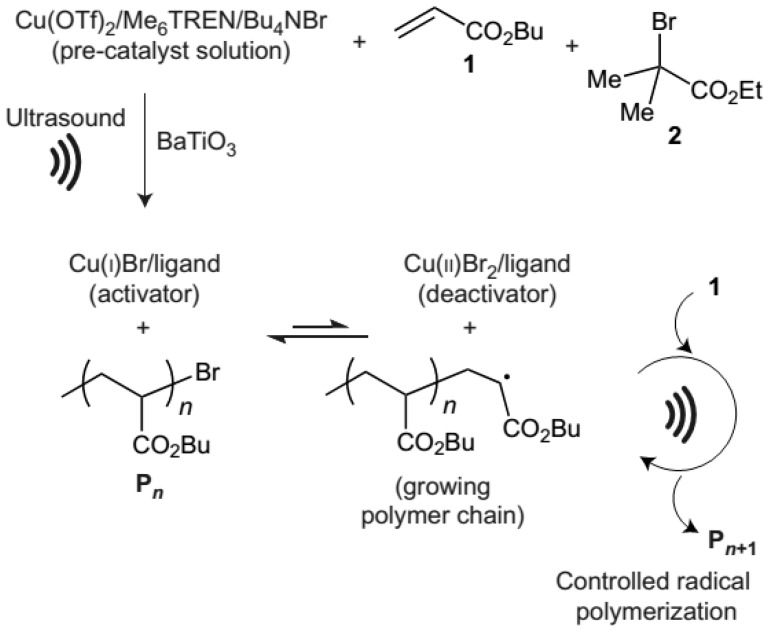
Mechanism of ultrasound-induced ATRP of BA 1 starting from the alkyl halide initiator 2 with the use of BaTiO_3_ piezoelectric nanoparticles [48]. Reprinted with permission from Springer Nature. Copyright 2016.

After the series of experiments, the predominant role of BaTiO_3_ in providing electrons to reduce Cu^II^ was concluded. Thus, the mechanism of ultrasound-mediated polymerization covers the initiating step, including sonochemical reduction of copper(II) in the catalyst/precursor mixture copper(II) triflate/*N*,*N*,*N*′,*N*′,*N*″,*N*″-hexamethyl(tris(aminoethyl)amine)/tetrabutylammonium bromide (Cu(OTf)_2_/Me_6_TREN/Bu_4_NBr) at the interface of the piezoelectric nanoparticle. It results in formation of the active form of catalyst, and thus starts the polymerization from the ethyl α-bromoisobutyrate (EBiB) initiator. The polymeric chain grows by successive additions of BA monomer (Figure 2) [48].

A switchable, controlled approach of mechanically induced polymerization was also used through stopping and restarting the process by switching off and on the ultrasound agitation, respectively. The poly(butyl acrylate) (PBA) macromolecules received retained all properties of materials made by conventional ATRP, including a linear increase in the polymer molecular weight and consistently low polydispersity [48].

**Figure 3:**
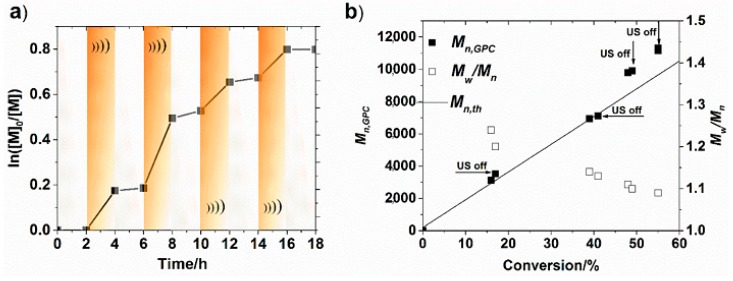
Temporally controlled mechano-ATRP approach in the polymerization of methyl acrylate (MA): (**a**) first-order kinetics plot, (**b**) evolution of molecular weight and dispersity of prepared polymers [41]. Reprinted with permission from American Chemical Society. Copyright 2017.

In comparison to the initial work in the field of mechano-ATRP, Matyjaszewski et al. go a step further, primarily by using only a small amount of catalyst (145 ppm) while maintaining the controlled characteristics of an ATRP process. Moreover, that investigation was focused on a detailed cognition of BaTiO_3_. NPs proved that mechanochemically-mediated polymerization is regulated by a few crucial factors. The more important covers dielectric constants (ε) of the transducers. It is obvious that a larger dielectric constant provides more effective electron transfer to a copper catalyst. Other factors include the crystal structures and particle sizes of the transducers. They were investigated by the use of a different type of BaTiO_3_: cubic and tetragonal with the size in the range 50–200 nm. Considering the crystal structure of NPs, the stronger piezoelectric effect was shown by tetragonal type of transducers, which is strongly connected with the highly distorted characteristic of BaTiO_3_ crystals and the above-mentioned dielectric constant [41]. Tetragonal BaTiO_3_ crystals have a significantly higher dielectric constant ε ~3000 than the cubic phase ε ~500 [63,64]. Examining the effect of the size of the nanoparticles, the smaller transducers improve the mechanoelectric conversion, which follows from a larger surface/interface effect and higher surface-to-volume ratios. The loading of NPs was also examined. Logically, a higher transducer loading provided faster polymerization due to a higher concentration of radicals. To avoid an aggregation and precipitation of BaTiO_3_ NPs in the solution, the work presents influence of modification of NPs by poly(methyl methacrylate) (PMMA) chains. The use of a modified mechano-electro transducer increases the electron transfer efficiency; thus, a reduction of an activator, stabilizes the nanoparticles and keeps them uniformly dispersed. As with all external stimuli, the characteristic feature for ultrasound agitation is the ability to temporal control over polymerization. Additionally, a low concertation of the catalyst was crucial to a switchable characteristic of the process, because the opportunity of a quick loss of activator results from halted regeneration of the catalyst, and thus radical termination reactions. The experiments proved efficient control over the polymerization, shown by negligible evolution of monomer conversion, and thus, molecular weights in the absence of ultrasonication, and continuously growing of polymer chains after the switching on the mechanical force again (Figure 3). Considering temporal control as a significant aspect of the ultrasound-mediated polymerization, and other benefits arising from using ultrasound for the preparation of polymers, e.g., elimination of chemical reducing agents, the process is more cost-effective and has an easily-scalable reaction setup, this technique is valuable for future industrial applications due to the possibility of the synthesis of (co)polymers with predicable molecular weights [41].

Matyjaszewski et al. present the full range of different issues directly connected with the mechanochemically-induced ATRP influencing the high development of both type and loading of transducers, and the possibility of temporal control, what makes this technique the future approach in the preparation of complex architectures.

The same research group in a short time significantly evaluated the mechano-ATRP approach by the investigation of another piezoelectric in the context of decreasing the transducer loading. The reaction conducted with BaTiO_3_ required ~4.5 wt.% of NPs loaded. It was necessary due to a weak interaction between a mechano-electro transducer and deactivator form of the catalyst, which resulted in an inefficient electron transfer in the presence of insufficient NP concentration. This issue was solved by the use of zinc oxide (ZnO) NPs. Despite a smaller piezoelectric coefficient of ZnO compared to BaTiO_3_ NPs, an efficient mechanochemically-induced polymerization was carried out even at a 0.06 wt.% loading of ZnO NPs, which means it is 75-fold lower than in previous paper. It resulted from the smaller size of ZnO NPs, and in relation to stronger interaction of semiconductor nanoparticle with a catalyst complex, improving the local Cu^II^ concentration on the transducer’s surface [40].

**Figure 4:**
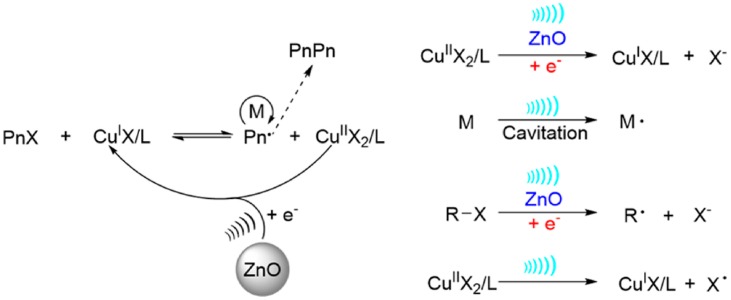
Proposed mechanism of mechano-ATRP with the use of ZnO NPs and mechanistic pathways for the activator regeneration [40]. Reprinted with permission from American Chemical Society. Copyright 2017.

In comparison to the first report in the mechano-ATRP [48], the authors considered two additional mechanistic pathways for the activator regeneration in the presence of a piezoelectric (Figure 4). Besides the ultrasound-mediated electron transfer from transducer to deactivated form of catalyst and the direct reduction of alkyl halide to form carbon radicals by ZnO NPs under ultrasound agitation, they also proposed the route for activation of the catalyst by formation of radicals from monomer/solvent under ultrasound cavitation, initiating new polymer chains, and a hemolytic cleavage of the tris(2-pyridylmethyl)amine (TPMA)/copper(II)-bromide bond [40].

The series of experiments clearly proved the predominant role of ZnO NPs as a mechanochemical reducing agent of Cu^II^ under the influence of sonication (pathway 1 in Figure 4) [40]. As in the case of use of BaTiO_3_ NPs [41], stabilization of ZnO NPs by different modifiers improves the electron transfer efficiency (modification with PMMA) and provides, e.g., a transparent characteristic of a polymerization media useful in various fields (octylamine-coated ZnO–OA–ZnO).

ZnO NP-mediated polymerization was successfully applied to polymerize a wide range of acrylate monomers, including MA, ethyl acrylate (EA), *tert*-buty acrylate (*t*BA), and BA-receiving polymers with low dispersity—predominantly molecular—making this approach versatile [40].

Further development of the approach went through the optimization of the reaction conditions, including catalyst loading (Cu^II^Br_2_/Me_6_TREN), initiator concentration, BaTiO_3_ NP loading, and various ultrasound power in the context of synthesis of precisely defined poly(methyl acrylate) (PMA) with low dispercities received in high conversions (>90%). The challenge to investigate mechno-ATRP in bulk conditions was also taken; however, without the solvent (dimethyl sulfoxide, DMSO) the polymerization did not occur, which indicates the limitation of the mechanically-mediated electron transfer without the sufficient amount of a solvent. However, the bulk polymerization of DMSO analogue (2-(methylsulfinyl)ethyl acrylate, MSEA) was successfully performed, but good control over the process was noticed only with high catalyst loading (1500 ppm). Nevertheless, it constitutes a step forward in mechanochemical induction in the ATRP process [42].

**Figure 5:**
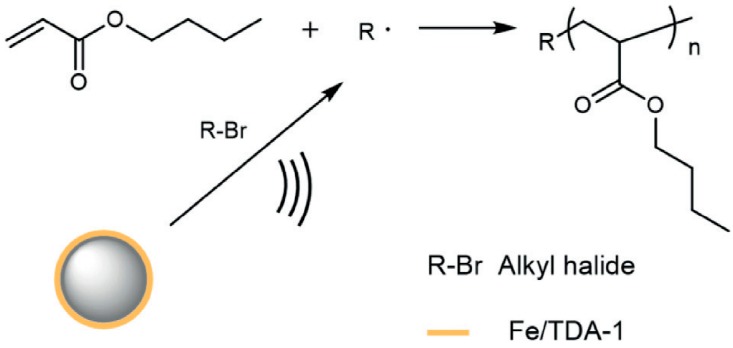
Mechanically controlled free-radical polymerization of BA [50]. Reprinted with permission from Wiley-VCH Verlag GmbH and Co. KGaA, Weinheim. Copyright 2019.

Inspired by searching for the cost-effective and industrially relevant methods in the synthesis of functional materials with remarkable mechanical properties and stabilities, the recent novelty among the piezocatalytic ATRP approach is focused on controlled free-radical (mechano-radical) polymerization and polymer crosslinking by a piezocatalytic reaction of different acrylate monomers. This investigation was implemented to develop the mechano-ATRP approach in the field of preparation for high molecular weight (HMW) polymers. Previously described procedures of mechano-ATRP provided polymers with relatively low molecular weights (Mw < 40,000). To reach this aim, the quite novel activity and higher radical concentration was achieved by an integration of iron(III) chloride hexahydrate/tris[2-(2-methoxyethoxy)ethyl]-amine (FeCl_3_·6H_2_O/TDA-1) complex with ZnO NPs. The proposed mechanism assumes that Fe^III^ located on the surface of piezoelectric facilitates the free-radical transfer during ultrasonic agitation, and thus, the piezocatalytic cleavage of alkyl halides occurs (Figure 5). It constitutes a different mechanism than in previous reports, where the predominant role of piezoelectric was to provide electrons to reduce Cu^II^ [50].

The application of Fe^III^ in connection with ZnO allows for the mechanically controlled free-radical polymerization and crosslinking in a one-pot fashion, and thus, more efficient consumption of ultrasound energy, making a mechanoradical polymerization potentially applicable and compatible with many conventional methods [50].

## 3. Ultrasonication-Induced ATRP in Homogenous and Dispersed Media

**Figure 6:**
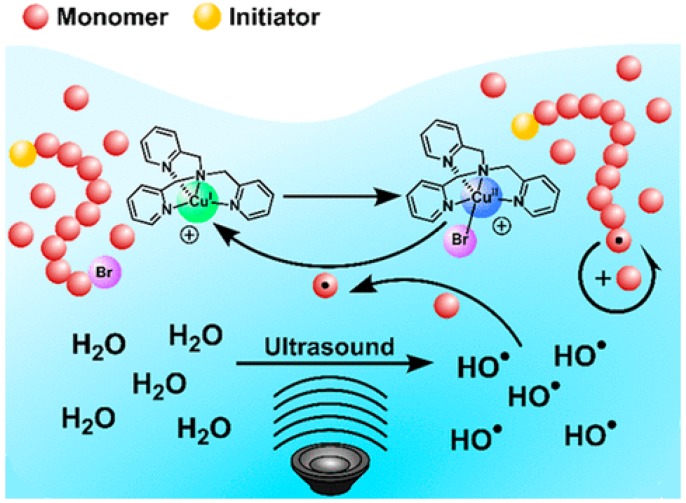
Mechanism of ultrasonication-induced ATRP [43]. Reprinted with permission from American Chemical Society. Copyright 2018.

The breakthrough in the field of application of an ultrasonic agitation in controlled polymerization is undoubtedly the procedure of sono-ATRP in aqueous media (Table 2). Comparing it to piezocatalytic approach, that application of an ultrasonic force was characterized by a different mechanism rather than a mechanochemically-induced solution, and it allows one to avoid the use of additional chemical reducing agents; e.g., piezoelectric. The main factor leading to create an activator are hydroxyl radicals produced due to the acoustic cavitation when the ultrasonic wave propagates through aqueous media. The resulting radicals are precursors to forming carbon radicals by a reaction with vinyl monomers and initiating a polymerization by direct reduction of Cu^II^ (Figure 6). To maintain control in aqueous ATRP, it is important to use a halide salt (e.g., sodium bromide (NaBr)) [43]. The predominant role of NaBr relies on reducing the dissociation of bromide anions from the deactivating species, and thus increasing its concentration, as well as minimizing hydrolysis of a brominated form of an initiator or growing polymer chain [38,43].

It was successfully applied to polymerizing a few water soluble monomers, including oligo(ethylene oxide) methyl ether methacrylate (OEOMA) and 2-hydroxyethyl acrylate (HEA), resulting in precisely defined homo and block (co)polymers using only ppm amounts of copper catalyst (going down to 40 ppm). Moreover, similar to the mechanochemical method, ultrasound agitation can temporarily take control over the polymer’s chain growth by modulation of forming the hydroxyl radicals by switching the ultrasonication between off and on stages, and thus stopping and restarting the activator regeneration and polymerization process. Going towards more specialized materials, sono-ATRP was implemented to prepare highly-functional biopolymers—DNA-based biohybrid polymerizing OEOMA providing a polymer with a low dispersity (*M*_w_/*M*_n_ = 1.41), and clearshifts in molecular weight of the DNA-polymer hybrid shifted to a higher range from the original DNA macroinitiator [43].

Instead of the low frequency ultrasonic agitation used in the both mechanically and ultrasonically induced reaction described above, Collins et al. applied high frequency ultrasonication (490 kHz) to polymerize HEA [51]. This treatment was intended to avoid the strong shear forces generated by low frequencies that result in polymer degradation, and thus, limitations in macromolecules’ sizes [65]. As in previous work [43], the proposed mechanism of reduction of the deactivator to active form of catalyst is based on the formation of radicals while the ultrasonic agitation flows through the aqueous media. However, besides the hydroxyl radicals formed through direct pyrolysis of solvent, the source of radicals was extended to hydrogen radicals (formed as a hydroxyl species), and an active form resulted from H-abstraction or radical addition within organic molecules. The main pathway to generate an active form of a copper catalyst is the formation of monomer-based radicals from direct pyrolysis under the influence of cavitation or by reaction with ultrasonic wave-generated hydroxyl/hydrogen radicals. That results in the formation a small amount of brominated by-products. Furthermore, the proposed reaction pathways also includes the direct reduction of Cu^II^ by hydrogen radicals, forming H–Br [51]. This high-frequency, ultrasound-based approach provided the poly(2-hydroxyethyl acrylate) (PHEA) macromolecules with a precisely defined structure, proven by the low dispersity and high conversion (>90%) received in a short period of time (<60 min) [51].

**Figure 7:**
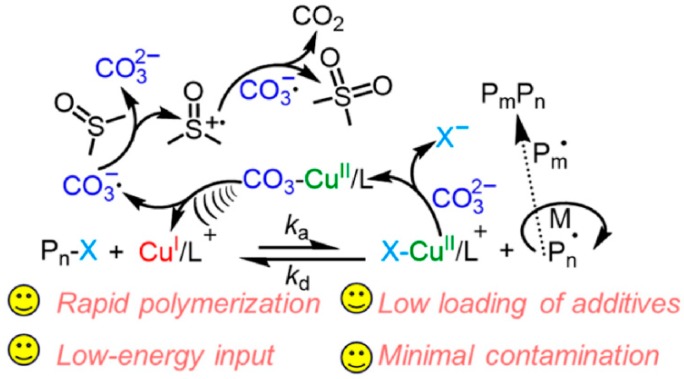
Mechanism of ultrasonication-induced ATRP with catalytic activation-mediated by carbonates [52]. Reprinted with permission from American Chemical Society. Copyright 2019.

Expanding sonochemical radical formation in controlled polymerization to a wider range of monomer types rather than water-soluble molecules, Matyjaszewski et al. developed sono-ATRP in the polymerization of hydrophobic monomers by using an organic solvent. This approach met the problems encountered in previous efforts in both mechano and sono-ATRP; namely, these procedures were characterized by contamination as new chains were initiated by residual piezoelectric nanoparticles or hydroxyl radicals, respectively. Replacing water medium with organic solvent, and thus, losing the ability to generate hydroxyl radicals by ultrasonic agitation, it was necessary to introduce the intermediary factor, leading to the formation of active species of a catalyst under the influence of ultrasound. Inspired by the fact that the labile copper(II)–OR bond in some copper(II)-based complexes undergoes homolytic cleavage, resulting in the formation of copper(I) and radical anions [66], a similar concept was introduced to homogenous sono-ATRP. Generation of ATRP activator was mediated by carbonate salts (sodium carbonate, Na_2_CO_3_). The fundamental role of Na_2_CO_3_ in the presence of ultrasound and ATRP catalysts includes the formation of carbonate complex with a catalyst ((CO_3_)Cu^II^TPMA) followed by the homolytic cleavage, which generates the activator species and a carbonate radical anion (Figure 7) [52].

A similar concept, without using a carbonate as a mediator in ultrasonication-induced ATRP, however, covers polymerization mediated by sonochemically-induced electron transfer (SET) in the presence of free ligand Me_6_TREN in DMSO. DMSO plays a crucial role in this approach. Ultrasonication caused the generation of DMSO-based radicals. The resultant active species reacted with the excess of ligand (Me_6_TREN), which was a source of an electron for the reduction of Cu^II^ to Cu^I^, and thus, activated the polymerization. This method was successfully investigated in the polymerization of MA and methyl methacrylate (MMA), wherein the apparent molecular weight of the methacrylate was higher than the theoretical value, which can be connected with the lower activity of the secondary alkyl halides of initiator molecules than the tertiary alkyl halide group in growing chains due to the higher C–Br bond dissociation energy and poorer stabilization of the derived radicals. The polymerization of styrene (St) did not occur. The experiments with different ligands proved that TPMA and *N*,*N*,*N*′,*N*″,*N*″-pentamethyldiethylenetriamine (PMDETA) did not work in this reaction setup, suggesting that the DMSO molecule or DMSO-based radicals cannot directly reduce a deactivator species and emphasized the significance of Me_6_TREN in the reducing electron generation [53].

**Figure 8:**
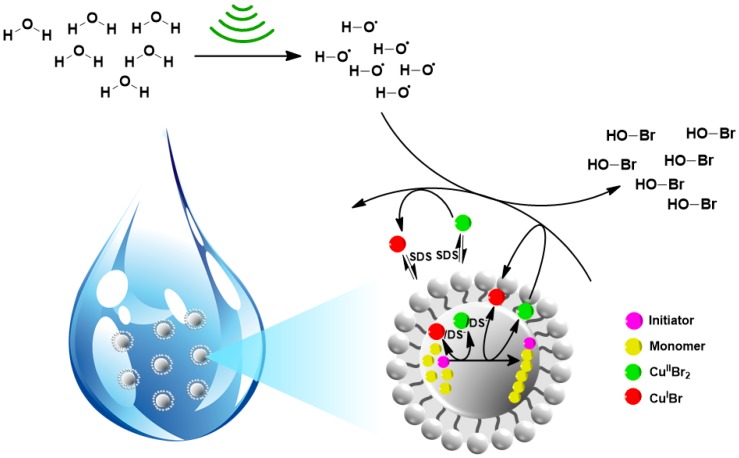
Ultrasound-mediated ATRP in a miniemulsion by interfacial and ion-pair catalysis [12]. Reprinted with permission from WILEY-VCH Verlag GmbH and Co. KGaA, Weinheim. Copyright 2019.

Inspired by the previous works in the field of ultrasonication-mediated ATRP activator generation and the increasing requirement for more environmentally friendly and industrially relevant approaches in macromolecules synthesis, our group most recently describes the concept, combining simultaneously, sono-ATRP in aqueous media and the possibility of polymerizing a hydrophobic monomer. As shown in Figure 8, the mechanism of sono-ATRP in miniemulsion media was considered in two major mechanisms—interfacial and ion-pair catalysis. The first one is predominant due to presence of 95% [67] of catalyst at the surface of monomer droplets, where it is reduced do Cu^I^, and thus, the polymerization is initiated. Another solution includes the combination of a reduced form of copper catalyst with sodium dodecyl sulfate, which acts as an anionic surfactant. It results in the formation of a neutral ion pair, Br–Cu^II^TPMA^+^/DS^−^, that possess the ability to activate the polymer inside of hydrophobic monomer droplets [12].

This approach was applied for both acrylates (BA, *t*BA) and methacrylates (MMA), resulting in the preparation of precisely defined homo and copolymers. A temporal control experiment was successfully conducted, showing that even a long induction period polymerization miniemulsion setup can be efficiently reinitiated without any loss in well-controlled polymerization characteristics (maintaining low dispersity and preservation of chain-end functionality) [12]. Taking into account the promising experimental results, environmentally friendly aspects, and easily scalable reaction setup, this solution constitutes a perfect alternative for an industrial application, and undoubtedly is the base for further development in ultrasound-mediated, controlled polymerization.

## 4. Future Prospective

Ultrasonication, as a versatile type of external stimuli, was applied in the ATRP in two different ways. Initially, it was implemented with connection to piezoelectric nanoparticles to cause mechano-induced electron transfer; therefore, continuously regenerating the activator species. The method was evaluated by eliminating the necessity of using transducers in the reaction setup and creating a possibility to polymerize a hydrophilic monomer. The quite different mechanism assumes the generation of radicals (from solvent, monomer, or ligand) due to the acoustic cavitation when the ultrasonic wave propagates through aqueous media. The breakthrough in the field of the use of an ultrasonic agitation in ATRP is the miniemulsion approach. It combines several significant features necessary for industrial use. From the point of view of “green chemistry”, it avoids using additional chemical compounds, eliminating the byproduct and additional step of purification of the final product, and thus, minimizing energy requirements. Moreover, it also does not use organic solvents due to water being used as a reaction medium, and simultaneously, it provides the polymerization of hydrophobic monomers. This method constitutes a strong future prospect for the use of an ultrasonic agitation in the ATRP method. All the ultrasonication-induced ATRP solutions are compatible with temporal control over polymerization, examined by turning on and off ultrasonic agitation, to start and stop the synthesis, respectively. Future efforts in this field should be directed toward the use of temporally-controlled sono-ATRP in the preparation of more complex architectures with highly predictable molecular weights and versatile properties. Considering all achievements in ultrasound-mediated controlled polymerization, numerous aspects for future development to come to mind. Undoubtedly, the main perspective is heading toward using this technique to prepare complex architectures; e.g., stars, brushes, bottlebrushes, and ultra-high MW polymers with specialized applications. Until now, DNA-based bioconjugates were successfully prepared by ultrasonication-induced ATRP in water, which is a perfect base for the further development in the implementation of ultrasound in the synthesis of biohybrids of polymers with other biomacromolecules. Despite the use of a small amount of catalyst, the challenge in ultrasonication-mediated ATRP is also minimizing the concentration of catalyst to an ultra-low ppm level.

External control by ultrasound represents an efficient, environmental, and cost-effective alternative to conventional ATRP; in particular, using a chemical reducing agent. It is expected to be the future solution for currently challenging targets in the field of the preparation of specialized polymer materials.

## Figures and Tables

**Table 1:** Mechanically controlled atom transfer radical polymerization (ATRP).

Initiator	Monomer	Catalyst Complex	Piezoelectrics	Solvent	Temp. (°C)	Ultrasound Frequencies (kHz)	Catalyst Concentration		Ref.
							ppm (Catalyst/Monomer)	ppm (by Weight)	
EBiB	BA	Cu^II^(OTf)_2_/Me_6_TREN ^a^	BaTiO_3_	DMF	15–25 °C	20	10,042	14,475	[48]
EBiB	MA	Cu^II^Br_2_/TPMA	BaTiO_3_	DMSO	R.T.	40	150	172	[41]
EBiB	MA	Cu^II^Br_2_/TPMA	ZnO	DMSO	R.T.	40	75–150	174 ^b^	[40]
EBiB	EA	Cu^II^Br_2_/TPMA	ZnO	DMSO	R.T.	40	150	–	[40]
EBiB	*t*BA	Cu^II^Br_2_/TPMA	ZnO	DMSO	R.T.	40	150	–	[40]
EBiB	BA	Cu^II^Br_2_/TPMA	ZnO	DMSO	R.T.	40	150	–	[40]
EBiB	MMA	Cu^II^Br_2_/TPMA	ZnO	DMSO	R.T.	40	150	–	[40]
PMA-Br	MA	Cu^II^Br_2_/TPMA	ZnO	DMSO	R.T.	40	97	95	[40]
EBiB	MA	Cu^II^Br_2_/Me_6_TREN	BaTiO_3_	DMSO	50 °C	40	100–200	172	[42]
EBiB	MSEA	Cu^II^Br_2_/Me_6_TREN	BaTiO_3_	DMSO	50 °C	40	150–1500	–	[42]
EBiB/MBiB/MBP/MBAc/mPEG bromoisobutyrate 5000	BA	Fe^III^Cl_3_·6H_2_O/TDA-1	ZnO	DMF/DMSO/NMP	20–30 °C	40	10,000	9730 ^c^	[50]
EBiB	*t*BA	Fe^III^Cl_3_·6H_2_O/TDA-1	ZnO	DMF/DMSO/NMP	20–30 °C	40	10,000	9730	[50]
EBiB	MA	Fe^III^Cl_3_·6H_2_O/TDA-1	ZnO	DMF/DMSO/NMP	20–30 °C	40	20,000	14,609	[50]
EBiB	MMA	Fe^III^Cl_3_·6H_2_O/TDA-1	ZnO	DMF/DMSO/NMP	20–30 °C	40	20,000	14,029	[50]
EBiB	EA	Fe^III^Cl_3_·6H_2_O/TDA-1	ZnO	DMF/DMSO/NMP	20–30 °C	40	10,000	10,933	[50]
EBiB	EHMA	Fe^III^Cl_3_·6H_2_O/TDA-1	ZnO	DMF/DMSO/NMP	20–30 °C	40	10,000	7630	[50]
PMMA-Br	BA	Fe^III^Cl_3_·6H_2_O/TDA-1	ZnO	DMF/DMSO/NMP	20–30 °C	40	150	–	[50]
EBiB	MA, EGDMA	Fe^III^Cl_3_·6H_2_O/TDA-1	ZnO	DMF/DMSO/NMP	20–30 °C	40	20,000	14,609	[50]
EBiB	HEA	Fe^III^Cl_3_·6H_2_O/TDA-1	ZnO	DMF/DMSO/NMP	20–30 °C	40	10,000	10,212	[50]

^a^ Bu_4_NBr as a supporting electrolyte; ^b^ for the reaction condition with 150 ppm (catalyst/monomer) of catalyst; ^c^ for the reaction with the use of EBiB as an initiator.

**Table 2:** Ultrasound-mediated ATRP in homogenous and dispersed media.

Initiator	Monomer	Catalyst Complex	Solvent	Temp. (°C)	Ultrasound Frequencies (kHz)	Catalyst Concentration		Ref.
						ppm (Catalyst/Monomer)	ppm (by Weight)	
PEG_2k_-BPA	OEOMA_500_	Cu^II^Br_2_/TPMA ^a^	H_2_O	R.T.	40	40–4000	39 ^b^	[43]
PEG_2k_-BPA	OEOMA_500_	Cu^II^Br_2_/TPMA ^a^	H_2_O/EtOH	R.T.	40	120–2000	40 ^b^	[43]
PEG_2k_-BPA	HEA	Cu^II^Br_2_/TPMA ^a^	H_2_O	R.T.	40	200–500	312 ^c^	[43]
P(OEOMA_500_)-Br	OEOMA_500_	Cu^II^Br_2_/TPMA ^a^	H_2_O	R.T.	40	3800	58	[43]
DNA-iBBr	OEOMA_500_	Cu^II^Br_2_/TPMA ^d^	H_2_O	R.T.	40	1000	–	[43]
HEBriB	HEA	Cu^II^Br_2_/Me_6_TREN	H_2_O	R.T.	490	50–1000	8–153	[51]
EBiB	MA	Cu^II^Br_2_/TPMA ^e^	DMSO	R.T.	40	150	178 ^f^	[52]
EBiB	MA	Cu^II^Br_2_/Me_6_TREN	DMSO	50 °C	40	150	177	[53]
EBiB	MA	Cu^II^Br_2_/TPMA	DMSO	50 °C	40	150	–	[53]
EBiB	MA	Cu^II^Br_2_/PMDETA	DMSO	50 °C	40	150	–	[53]
EBiB	MMA	Cu^II^Br_2_/Me_6_TREN	DMSO	50 °C	40	150	–	[53]
EBiB	St	Cu^II^Br_2_/Me_6_TREN	DMSO	50 °C	40	150	–	[53]
EBIB	BA	Cu^II^Br_2_/TPMA ^g^	H_2_O ^h^	65 °C	40	717	192–218	[12]
EBiB	MMA	Cu^II^Br_2_/TPMA	H_2_O	65 °C	40	717	290	[12]
EBPA	MMA	Cu^II^Br_2_/TPMA	H_2_O	65 °C	40	717	154	[12]
PBA-Br	BA	Cu^II^Br_2_/TPMA	H_2_O	65 °C	40	717	150	[12]
EBiB	*t*BA	Cu^II^Br_2_/TPMA	H_2_O	65 °C	40	717	151	[12]

^a^ NaBr as a supporting electrolyte; ^b^ for the reaction with 400 ppm (catalyst/monomer) of catalyst; ^c^ for the reaction with 500 ppm (catalyst/monomer) of catalyst; ^d^ NaCl as a supporting electrolyte; ^e^ with the use of Na_2_CO_3_; ^f^ for 0.05 wt.% of Na_2_CO_3_; ^g^ NaBr as a supporting electrolyte, sodium dodecyl sulfate (SDS) as a surfactant and hexadecane as a cosurfactant; ^h^ miniemulsion medium.
